# Supplementary material for: Resolving the full spectrum of human genome variation using Linked-Reads
Source: Genome Res. 2019 Apr;29(4):635–45. doi: 10.1101/gr.234443.118 (PMC6442396; doi:10.1101/gr.234443.118)
Supplement: Supplemental Material [file supp_gr.234443.118_Supplemental_longranger-2.2.zip › longranger-2.2.tar.bz2!/lr2.2-public/lib/stan/cmdstan/stan/doc/api/html/classstan_1_1optimization_1_1_b_f_g_s_update___h_inv.html]

Stan: stan::optimization::BFGSUpdate\_HInv< Scalar, DimAtCompile > Class Template Reference


|  |  |
| --- | --- |
|  | Stan  2.5.0  probability, sampling & optimization |


- Main Page
- Related Pages
- Namespaces
- Classes
- Files

- Class List
- Class Index
- Class Hierarchy
- Class Members

All Classes Namespaces Files Functions Variables Typedefs Enumerations Enumerator Friends Macros Pages

- stan
- optimization
- BFGSUpdate\_HInv

Public Types |
Public Member Functions |
List of all members

stan::optimization::BFGSUpdate\_HInv< Scalar, DimAtCompile > Class Template Reference

`#include <bfgs_update.hpp>`

|  |  |
| --- | --- |
| Public Types | |
| typedef Eigen::Matrix< Scalar,   DimAtCompile, 1 > | VectorT |
|  | |
| typedef Eigen::Matrix< Scalar,   DimAtCompile, DimAtCompile > | HessianT |
|  | |

|  |  |
| --- | --- |
| Public Member Functions | |
| Scalar | update (const VectorT &yk, const VectorT &sk, bool reset=false) |
|  | Update the inverse Hessian approximation. More... |
|  | |
| void | search\_direction (VectorT &pk, const VectorT &gk) const |
|  | Compute the search direction based on the current (inverse) Hessian approximation and given gradient. More... |
|  | |

## Detailed Description

### template<typename Scalar = double, int DimAtCompile = Eigen::Dynamic> class stan::optimization::BFGSUpdate\_HInv< Scalar, DimAtCompile >

Definition at line 10 of file bfgs\_update.hpp.

## Member Typedef Documentation

template<typename Scalar = double, int DimAtCompile = Eigen::Dynamic>

typedef Eigen::Matrix<Scalar,DimAtCompile,DimAtCompile> stan::optimization::BFGSUpdate\_HInv< Scalar, DimAtCompile >::HessianT

Definition at line 13 of file bfgs\_update.hpp.

template<typename Scalar = double, int DimAtCompile = Eigen::Dynamic>

typedef Eigen::Matrix<Scalar,DimAtCompile,1> stan::optimization::BFGSUpdate\_HInv< Scalar, DimAtCompile >::VectorT

Definition at line 12 of file bfgs\_update.hpp.

## Member Function Documentation

template<typename Scalar = double, int DimAtCompile = Eigen::Dynamic>

|  |  |  |  |
| --- | --- | --- | --- |
| void stan::optimization::BFGSUpdate\_HInv< Scalar, DimAtCompile >::search\_direction | ( | VectorT & | *pk*, |
|  |  | const VectorT & | *gk* |
|  | ) |  | const |

inline

Compute the search direction based on the current (inverse) Hessian approximation and given gradient.

Parameters
:   |  |  |  |
    | --- | --- | --- |
    | [out] | pk | The negative product of the inverse Hessian and gradient direction gk. |
    | [in] | gk | Gradient direction. |

Definition at line 56 of file bfgs\_update.hpp.

template<typename Scalar = double, int DimAtCompile = Eigen::Dynamic>

|  |  |  |  |
| --- | --- | --- | --- |
| Scalar stan::optimization::BFGSUpdate\_HInv< Scalar, DimAtCompile >::update | ( | const VectorT & | *yk*, |
|  |  | const VectorT & | *sk*, |
|  |  | bool | *reset* = `false` |
|  | ) |  |  |

inline

Update the inverse Hessian approximation.

Parameters
:   |  |  |
    | --- | --- |
    | yk | Difference between the current and previous gradient vector. |
    | sk | Difference between the current and previous state vector. |
    | reset | Whether to reset the approximation, forgetting about previous values. |

Returns
:   In the case of a reset, returns the optimal scaling of the initial Hessian approximation which is useful for predicting step-sizes.

Definition at line 26 of file bfgs\_update.hpp.

---

The documentation for this class was generated from the following file:

- src/stan/optimization/bfgs\_update.hpp


---

[ Stan Home Page ]

*© 2011–2014,
Stan Development Team.*
